# Heavy Metal Accumulation in Freshwater Fish: The Role of Species, Age, Gender, and Parasites

**DOI:** 10.1007/s00128-025-04068-z

**Published:** 2025-06-10

**Authors:** Tímea Brázová, Yaroslav Syrota, Mikuláš Oros, Dalibor Uhrovič

**Affiliations:** 1 Institute of Parasitology, Slovak Academy of Sciences, 04001 Košice, Slovakia; 2 I. I. Schmalhausen Institute of Zoology NAS of Ukraine, Bogdan Khmelnytsky Street, 15, Kyiv, 01054 Ukraine; 3 Department of Zoology, Institute of Biology and Ecology, P. J. Šafárik University, Šrobárova 2, 040 01 Košice, Slovakia

**Keywords:** Fish, Pollution, Bioindicator, Parasites, Heavy metals, Slovakia

## Abstract

The present study investigates the distribution of cadmium, chromium, and mercury levels in sediments, plants, and fish from Zemplínska Šírava, a historically contaminated reservoir in Slovakia. Using fish as bioindicators, we analyzed the impact of factors such as age, gender, ecological group, and parasite burden on heavy metal accumulation. Results revealed that mercury was highest in fish muscle, particularly predatory species, whereas chromium and cadmium were predominantly found in liver tissues. Chromium accumulation was influenced by gender, with females showing higher levels. Older fish exhibit greater cadmium accumulation in liver tissue. Parasite burden was associated with elevated mercury levels in fish muscle. Notably, the cestode *Caryophyllaeus laticeps* had significantly higher chromium levels than its host, freshwater bream (*Abramis brama*). This study highlights the important role of fish and parasites in monitoring aquatic contamination and illustrates the complex interplay of biological and environmental factors in heavy metal bioaccumulation.

**Supplementary Information:**

The online version contains supplementary material available at 10.1007/s00128-025-04068-z.

## Introduction

Due to its persistent and toxic nature, heavy metal pollution in aquatic environments has become a significant environmental issue. This pollution harms water quality, biodiversity, and the overall health of ecosystems. Metals such as cadmium (Cd), chromium (Cr), and mercury (Hg) enter aquatic systems through industrial discharges, agricultural runoff, and natural sources. Once they enter these systems, they accumulate in sediments, plants, and aquatic organisms (Dehkordi et al. 2024). One effective method for detecting environmental pollution is to use animals from polluted areas as bioindicators. These organisms accumulate contaminants from their surroundings or adapt to adverse conditions, providing valuable information about the chemical status of their habitat (Hamza-Chaffai 2014).

Fish, which occupy various trophic levels in aquatic food webs and interact closely with water and sediment, are susceptible to heavy metal contamination, making them reliable bioindicators (Tenji et al. 2020). In natural ecosystems, fish are exposed to numerous endogenous and exogenous factors alongside pollutants (Sures and Nachev 2022). To understand the compounded stressors in nature, it is essential to analyze the complex impact of various factors, including fish species, size, age, feeding habits, pathogens, and environmental conditions on the bioaccumulation of heavy metals in fish (Pillet et al. 2021).

Ecotoxicological studies have proven that intestinal fish endohelminths are very effective in indicating various types of pollution (e.g. heavy metals and polychlorinated biphenyls), with concentrations in their tissues several times higher than in the hosts (Brázová et al. 2015; Sures et al. 2017; Oros et al. 2023), so they can reflect local pollution with high sensitivity.

The investigated Zemplínska Šírava reservoir is historically considered one of the most contaminated hotspots in Europe due to polychlorinated biphenyls (Šalgovičová and Zmetáková 2006). Currently, no data is available on heavy metal concentrations in the biota of the reservoir. Thus, this study examines the levels of heavy metals in different fish species and the factors that influence these levels, such as species, matrices, age, gender, and parasites.

## Material and Methods

From 2019 to 2021, 16 sediment samples of approximately 500 g from the Zemplínska šírava reservoir (For detailed information about the locality, see Oros et al. 2023) were taken with a dip sampler (TeleScoop, stainless steel V2A, capacity of 1000 ml), according to STN ISO 5667–12. Additionally, 19 samples of rooted aquatic plants (*Mentha aquatica*, *Carex acutiformis*, *Potamogeton natans*, *Myrriophylus spicatum*) were collected from the same sampling sites as sediments. Sediment and plants were stored separately in plastic bags at − 20 °C until further analysis. 101 fish of seven species (Table 1) were sampled annually in the Zemplínska Šírava reservoir by electrofishing and fishing rods under a permit (Nos. 62/2020 and 30/2021) issued by the Ministry of Environment of the Slovak Republic. The animal study was reviewed and approved by the Ethics Committee of the Institute of Parasitology of the Slovak Academy of Sciences, which also approved the implementation of the project under approval No. 1/2020/PaU. All methods used in the present study were carried out by relevant guidelines and regulations (Decree of the Ministry of the Slovak Republic no. 381/2018 Coll. and Act No. 216/2018 Coll. about fishing). Fishes were killed by severing the spinal cord, then weighted and standard length measured. The muscles and livers (hepatopancreas in cyprinids) were removed using stainless steel instruments and stored at − 20 °C frozen until further heavy metal analysis. The age of the fish was determined according to Carbonara and Follesa (2019) based on the scales collected from the part above the lateral line using a Zeiss Stemi 508 stereomicroscope with an Axiocam ERc5s camera. The age of Wels catfish was determined from the vertebra and according to Alp et al. (2011). The biological parameters of fish are presented in Table 1.

**Table 1:** Biometric parameters of fish (standard length, weight, age)

Hosts and parasites	Length (cm)	Weight (g)	Age	Prevalence, % (95% CI)	Intensity (range)
Piscivorous					
Asp (*Leuciscus aspius* (L.)) (N = 6)	57–75	2.9–3.5	3.6		
*Aspidogaster sp.*				16.7 [0.8–58.9]	286
*Proteocephalus sp.*				16.7 [0.8–58.9]	21
European perch (*Perca fluviatilis* L.) (N = 15)	23–38	0.2	1.10		
*Cyathocephalus truncatus* (Pallas, 1781)				40.0 [19.1–66.8]	98.7 [1–291]
*Acanthocephalus lucii* (Müller, 1776)				40.0 [19.1–66.8]	4.7 [1–10]
Northern pike (*Esox lucius* L.) (N = 4)	50–68	3.3	2.4	No helminths found	
Pike-perch (*Sander lucioperca*) (N = 7)	50–64	2.2	3.6		
*Cyathocephalus truncatus* (Pallas, 1781)				100.0 [62.3–100.0]	137.6 [15–608]
Wels catfish (*Silurus glanis* L.) (N = 6)	50–189	0.8–49	2.17		
*Glanitaenia osculata* (Goeze, 1782)				66.7 [27.13–93.7]	68.2 [20–100]
Omnivorous					
Frehwater bream* (Abramis brama* (L.)) (N = 48)	34–46	0.5–0.99	5.9		
*Caryophyllaeus laticeps* (Pallas, 1781)				58.3 [43.7–72.1]	15.4 [1–104]
Common carp (*Cyprinus carpio* L.) (N = 15)	29–94	0.35–13	2.9		
*Atractolytocestus huronensis* Anthony, 1958				73.3 [46.6–90.3]	47.3 [1–210]
*Khawia sinensis* Hsü, 1935				13.3 [2.4–39.7]	3.5 [3–4]

Prevalence (with 95% Sterne’s confidence intervals in square brackets) and mean intensity (with minimum and maximum values in square brackets) of found helminth taxa across seven fish species. The number of individuals examined for each fish species is denoted by ‘N’ following their scientific names

The intestines of fish were examined for parasites. The parasites found in the intestines were washed in saline, counted, and then preserved in either formaldehyde (4%) or ethyl alcohol (70%) for identification. We used bream-*Caryophyllaeus laticeps* as a model for comparing heavy metal accumulation in fish and parasites due to the lack of significant biomass of other parasitic individuals available for the analytical survey. The prevalence and intensity of gastrointestinal parasites are presented in Table 1. The scientific and common names of the fish were taken from the Fish-Base database (Froese and Pauly 2024).

All sediments, plants, and biological tissue samples were digested in a microwave oven (Ethos One, Milestone, Italy) for metal analysis. Digestion was accomplished in a rotating 10-position sample carousel with Teflon digestion vessels. The total concentration of heavy metals in sediments was determined by Flame-AAS (ZEEnit 700P, Analytik Jena, Germany). Approximately 0.5 g of plants, muscle, and liver and 0.1–0.3 g of parasite tissue were acid-digested by 7 ml of concentrated 65% HNO_3_ and 1 ml of 30% H_2_O_2_ (both Suprapur, Merck, Germany). After cooling, the solutions were transferred into 50 mL volumetric flasks, and diluted with ultrapure water, which was stored at + 4 °C until further metal analysis. All calibration and blank solutions were prepared with high-purity Milli-Q water. Cd and Cr concentrations were measured by the GFAAS technique using ZEEnit 700P (Analytik Jena, Germany). Argon of 99.998% purity was used as an inert gas. To determine the Hg concentrations, the samples were analyzed using hydride generations atomic absorption spectrophotometry (HG AAS). To assess the AAS analysis accuracy, the certified reference materials LGC6187 (river sediment), NCS ZC 73032 (Celery), and TORT-2 (Lobster hepatopancreas) for the individual samples as standards were analyzed. Each determination was replicated three times. All results are expressed in mg kg^−1^ wet weight (w. wt.) to enable comparison with the maximum levels fixed by European legislation. The overall recovery rates (mean ± SD) of Cd, Cr, and Hg were 97 ± 1.5, 95 ± 2.3, and 93 ± 3.2, respectively. Blank samples, consisting of all laboratory reagents, were analyzed with every 10 samples.

Data analysis was conducted in R (R Core Team 2023) using the tidyverse package (Wickham et al. 2019) for data manipulation and visualization. Measurement values of mercury, chromium, and cadmium that fell below detection thresholds were imputed with random values ranging from the detection limit to 100 times lower. The detection limits were 0.00102 mg/kg for mercury, 0.00085 mg/kg for chromium, and 0.00077 mg/kg for cadmium. To maintain consistency, fixed seeds were assigned to generate random values: 46 for mercury, 89 for chromium, and 90 for cadmium. This imputed dataset was used exclusively for descriptive statistics and univariate tests. Outliers, defined as values more than two standard deviations from the mean, were removed before conducting univariate tests. Wilcoxon signed-rank tests were then used to evaluate differences in metal concentration between liver and muscle tissues, and Spearman’s correlation was used to assess associations between bioaccumulation levels in these tissues. Three censored regression models were fitted with the *brm* function from the brms package (Bürkner 2017) to examine factors influencing metal bioaccumulation. Dependent variables were selected based on prior univariate tests: for each metal, the tissue (muscle or liver) with the higher concentration was chosen for modeling. Lognormal distribution was adopted to account for right-skewed data, and fixed effects included age, gender, ecological groups, and helminth burden (helminth count per individual fish) as fixed effects. Year and fish species were incorporated as random effects. Missing values in the fixed effects were omitted before model fitting. The models’ diagnostics included MCMC trace plots, posterior predictive checks, and Leave-One-Out Cross-Validation. Additionally, collinearity among predictors was assessed. Prevalence and intensity of gastrointestinal helminths were calculated by Bush et al. (1997). Confidence intervals for prevalence were calculated following Rózsa et al. (2000) via the *epi.prev* function from the epiR package (Nunes et al. 2020). A zero-inflated negative binomial model examined the relationship between helminth intensity (dependent variable) and fish gender (fixed effect), accounting for random effects of species and year. In addition, Spearman’s correlation tested the association between heavy metal accumulation and parasites in bream and *Caryophyllaeus laticeps*, selected for their prominence in the sample and parasite biomass.

## Results and Discussion

We investigated the content of heavy metals (Cd, Cr, and Hg) in three ecosystem components. Comparing mercury accumulation in fishes, plants, and sediments, we found that fishes and sediments had the highest median concentrations (Table 2). In contrast, the highest median concentrations of chromium and cadmium were found in plants (Table 2). Aquatic plants absorb Cd and Cr from the sediments via roots and surface tissues (Kabata-Pendias 2010). These metals accumulate more efficiently in plants due to their higher bioavailability, uptake via ion transporters, and greater mobility within plant tissues (Benavides et al. 2005). Mercury, by contrast, binds more readily to organic matter in sediments, making it less bioavailable to plants but more likely to accumulate in animals after environmental transformations such as methylation (Lux et al. 2011). This may explain our results showing higher Cd and Cr levels in plants and higher Hg concentrations in fish.

**Table 2:** Comparative descriptive statistics of mercury, chromium, and cadmium accumulation in fishes, plants, and sediments (mg kg^−1^ w. wt)

	Hg			Cr			Cd		
	Fishes (N = 101)	Plants (N = 19, I = 0)	Sediments (N = 16, I = 0)	Fishes (N = 101)	Plants (N = 19, I = 0)	Sediments (N = 16, I = 6)	Fishes (N = 101)	Plants (N = 19, I = 0)	Sediments (N = 16, I = 8)
Mean	0.161	0.099	0.111	0.182	6.988	0.008	0.107	0.312	0.058
Median	0.124	0.083	0.114	0.113	3.842	0.002	0.083	0.167	0.010
SD	0.137	0.064	0.068	0.369	6.051	0.013	0.101	0.344	0.090

‘N’ indicates the total number of observations in the samples, while ‘I’ denotes the number of imputed values. For fishes, before calculating descriptive statistics for each metal, the average concentration per fish individual was evaluated, considering measurements from both liver and muscle tissues. In cases where only one type of tissue measurement (liver or muscle) was available, that singular measurement was included in the analysis

**Fig. 1:**
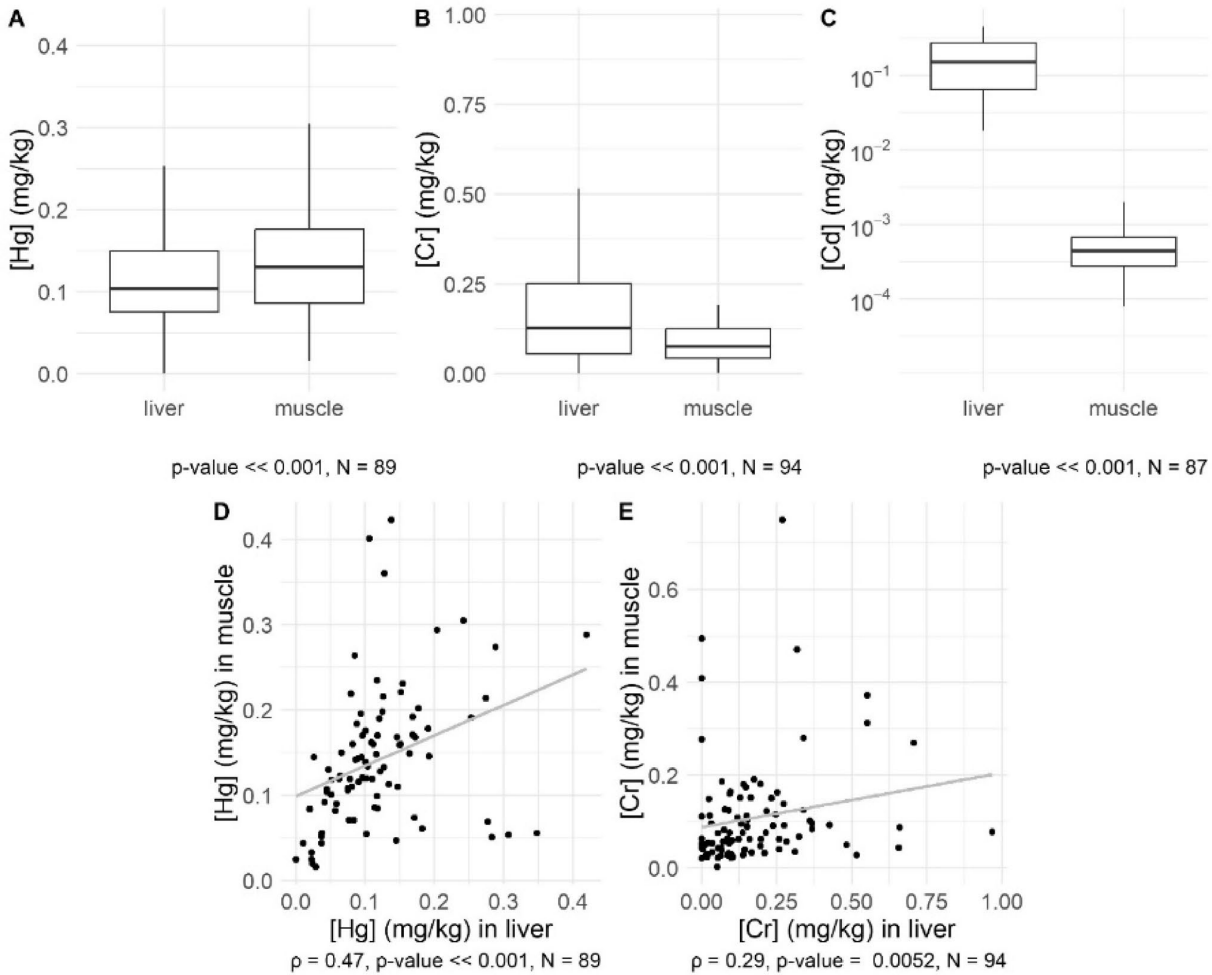
A comparative analysis of heavy metal concentrations across tissue samples of different fish species based on outlier-cleaned data. Panels (**A**), (**B**), and (**C**) represent the levels of mercury, chromium, and cadmium concentrations, respectively, shown via box plots to illustrate the interquartile ranges and medians and include results from the Wilcoxon signed-rank tests. Panels (**D**) and (**E**) show scatter plots that correlate the concentrations of mercury and chromium in the liver with their levels in muscle tissue, respectively, and include Spearman’s correlation test results. A correlation assessment between cadmium concentration in the liver and muscles was not conducted as the data for this metal contained too many imputed observations

We found statistically higher mercury concentrations in fish muscle than in livers (Table 3, Fig. 1a, d). Fish accumulate mercury through direct absorption from water and contaminated prey (Ali et al. 2019). While primarily found in detoxifying organs like the liver and kidneys, muscle tissue also serves as a significant reservoir, leading to prolonged bioaccumulation due to its limited detoxification capacity (Vieira et al. 2021). Golovanova (2008) identified the distribution order of mercury in fish as muscle, liver, intestine, spleen, brain, and gonads, attributing this to the high affinity of muscle proteins for mercury, explaining the higher Hg concentrations in muscles compared to the liver. The highest mercury concentrations were found in the muscles and liver of predatory Asp (*L. aspius*) (Table 4). As predatory fish consume smaller fish, mercury levels increase up the food chain, leading to higher concentrations in top predators. This has been documented in several studies (e.g., Squadrone et al. 2013; Borisov et al. 2023).

**Table 3:** Comparative descriptive statistics of mercury, chromium, and cadmium accumulation in muscle and liver tissues (mg kg^−1^ w. wt)

	Hg		Cr		Cd	
	Muscle (N = 100, I = 0)	Liver (N = 97, I = 1)	Muscle (N = 100, I = 0)	Liver (N = 97, I = 11)	Muscle (N = 100, I = 89)	Liver (N = 97, I = 9)
Mean	0.163	0.162	0.168	0.205	1.3 × 10^–3^	0.220
Median	0.136	0.110	0.065	0.129	4 × 10^–4^	0.165
SD	0.121	0.189	0.640	0.335	3.3 × 10^–3^	0.203

‘N’ indicates the total number of observations in the sample, while ‘I’ denotes the number of imputed values

**Table 4:** Concentrations (mg kg^−1^ w. wt) of Mercury (Hg), Chromium (Cr), and Cadmium (Cd) in the muscle and liver of different fish species

	Species	Muscle				Liver			
		Mean	Median	SD	N	Mean	Median	SD	N
Hg	*C. carpio*	0.0519	0.047	0.0281	15	0.0582	0.037	0.0532	15
	*L. aspius*	0.3383	0.338	0.2349	6	0.463	0.467	0.3601	6
	*A. brama*	0.155	0.143	0.0816	47	0.1308	0.104	0.1474	47
	*E. lucius*	0.2058	0.1995	0.0502	4	0.1232	0.1195	0.0465	4
	*P. fluviatilis*	0.1628	0.146	0.1039	15	0.1434	0.117	0.0741	13
	*S. glanis*	0.2288	0.2145	0.1484	6	0.3598	0.242	0.3686	5
	*S. lucioperca*	0.2213	0.196	0.1567	7	0.2539	0.253	0.1234	7
Cr	*C. carpio*	0.1051	0.076	0.0872	15	0.2579	0.143	0.2643	15
	*L. aspius*	0.0893	0.0855	0.0494	6	0.2307	0.1945	0.1684	6
	*A. brama*	0.27	0.093	0.9254	47	0.1503	0.093	0.1813	47
	*E. lucius*	0.0683	0.0505	0.0599	4	0.1593	0.1565	0.074	4
	*P. fluviatilis*	0.0744	0.057	0.0552	15	0.1748	0.152	0.1304	13
	*S. glanis*	0.0475	0.0485	0.0168	6	0.2158	0.258	0.0983	5
	*S. lucioperca*	0.0443	0.038	0.0224	7	0.5181	0.067	1.0785	7
Cd	*C. carpio*	0.0038	0.0006	0.0067	15	0.0886	0.063	0.1035	15
	*L. aspius*	0.0005	0.0005	0.0002	6	0.0892	0.0795	0.0713	6
	*A. brama*	0.0007	0.0004	0.0015	47	0.3446	0.323	0.2137	47
	*E. lucius*	0.0045	0.0028	0.0054	4	0.0067	0.0004	0.0128	4
	*P. fluviatilis*	0.0003	0.0003	0.0002	15	0.1401	0.137	0.081	13
	*S. glanis*	0.0019	0.0006	0.0035	6	0.114	0.06	0.1129	5
	*S. lucioperca*	0.0004	0.0004	0.0003	7	0.1215	0.12	0.1028	7

Contrarily to mercury, the observed concentrations of chromium and cadmium were higher in the liver of fishes than in muscle tissue, and the Wilcoxon paired test indicated a significant difference in both cases (refer to Fig. 1b and c). There was a weak positive correlation between chromium levels in muscle and liver tissues (see Fig. 1e). The highest median chromium concentration was found in the liver of the Wels catfish (Table 4). Additionally, cadmium was most accumulated in the liver of the bream (Table 4).

Due to its high metabolic activity, the liver plays a crucial role in detoxifying, redistributing, and transforming heavy metals (Zhao et al. 2012). It is one of the first organs to reflect environmental contamination and acts as a key site for metal metabolism due to the presence of binding proteins such as metallothioneins (MT). Several studies consistent with our results report high levels of Cd and Cr, in fish liver tissues (Zhao et al. 2012; Liu et al. 2015).

Eight helminth taxa were identified in the study. Notably, *C. truncatus* was found in two fish species, while northern pike (*E. lucius*) had no helminths (Table 1). Regression analysis showed no significant link between helminth count and fish gender (β = − 0.45, 95% CI: − 1.30, 0.44). Similarly, Oros et al. (2023) found no gender influence on the infection intensity of cestodes *Glanitaenia osculata* in Wels catfish.

It is well-documented that parasites can bioaccumulate heavy metals in higher amounts than fish or even have the ability to detoxify their infected hosts (Sures et al. 2017). The present study found that the observed mercury concentration in freshwater bream (*A. brama*) was higher than in the parasites (*C. laticeps*), while the observed chromium levels in *C. laticeps* were about three times higher than in the bream. However, no statistically significant associations were found between the concentrations of metals in the host and parasite tissue samples for these two elements (Table 5). Palíková et al. (2014) also found about 7 times lower Hg concentrations in the cestode *Ligula intestinalis* than in the bream (*Abramis brama*). Mercury generally accumulates in lower concentrations in parasites than in their hosts. This is probably because they excrete mercury through detoxification processes (Palíková and Baruš 2003) and perhaps because mercury strongly binds to muscle tissues, making it less bioavailable for parasites (Geeraerts and Belpaire 2010). Descriptive analysis of metal concentrations showed no statistically significant differences between infected and uninfected breams (Fig. S1), likely attributable to the low overall metal levels. Regression analysis (Table 6) revealed that the age, gender, and ecological group of the fish had no meaningful influence on mercury accumulation in muscle tissue. However, a higher helminth burden in the fish was associated with a small but meaningful increase in muscle mercury levels. One possible explanation for this correlation is that methylmercury may reduce the host’s immune response, leading to a higher incidence of parasitic infections (Sagerup et al. 2009). Chronic mercury exposure can weaken the immune defenses of fish, making them more susceptible to parasitic infections (El-Hak et al. 2022). In this study, however, the immune responses associated with toxins and parasites were not investigated in detail.

**Table 5:** Comparative descriptive statistics of mercury accumulation in muscle tissue and chromium in liver tissue of the host (*A. brama*) and parasite (*C. laticeps*) with the results of correlation analysis (mg kg^−1^ w. wt)

Metal	Matrix	Mean	Median	SD	Min	Max	Spearman’s correlation
Hg	*A. brama*	0.13	0.13	0.04	0.08	0.18	ρ = − 0.09, *p*-value = 0.74, N = 8
Hg	*C. laticeps*	0.04	0.04	0.02	0.01	0.07	
Cr	*A. brama*	0.23	0.13	0.20	0.04	0.66	ρ = 0.47, *p*-value = 0.17, N = 10
Cr	*C. laticeps*	0.56	0.38	0.58	0.13	2.04

**Table 6:** Summary report of regression models assessing the relationship between metal accumulation and factors of interest

Predictors	Hg		Cr		Cd	
	Estimates	CrI (95%)	Estimates	CrI (95%)	Estimates	CrI (95%)
Intercept	− 2.02	− 3.22 to − 0.84	− 1.72	− 4.48 to 1.10	− 5.35	− 7.52 to − 3.09
Age	0.02	− 0.02 to 0.07	0.07	− 0.15 to 0.28	**0.32**	**0.15–0.49**
Ecological group						
Carnivorous	(Ref.)		(Ref.)		(Ref.)	
Omnivorous	− 0.71	− 2.04–0.47	− 1.25	− 3.81–1.42	0.98	− 2.27–4.51
Total intensity	**2.4 × 10**^**–3**^	**10**^**–3**^**–3 × 10**^**–3**^	− 9.6 × 10^–4^	− 0.01–5 × 10^–3^	2.6 × 10^3^	− 1.9 × 10^–3^–0.01
Random effects						
σ2	0.44		2.10		1.61	
τ00	0.42 _fish species_		1.68 _fish species_		3.44 _fish species_	
	0.42 years		4.32 years		0.24 years	
ICC	0.66		0.74		0.70	
N	3 years		3 years		3 years	
	7 _fish species_		7 _fish species_		7 _fish species_	
Observations	100		95		93	
Marginal R^2^	0.388		0.500		0.509	
Conditional R^2^	0.566		0.504		0.542	

‘CrI’ denotes 95% credible intervals; ‘σ2’ is the model’s mean–variance of random effects; ‘τ00’ represents the random intercept variance; and ‘ICC’ is the intraclass correlation coefficient. Meaningful predictors are in bold

Age, ecological group, and helminth burden showed no association with chromium levels in liver tissue, but gender did, with females exhibiting meaningfully higher levels than males (Table 6). Female fish express higher levels of metallothioneins—proteins regulated by hormones like estrogen—that bind heavy metals and aid detoxification, leading to sex-specific differences (Monteiro et al. 2010). Increased metal accumulation in females supports reproductive demands, as they consume more food during spawning (Burger et al. 2007), which could explain the higher chromium concentrations in the livers of females than in males in the present study. Regarding cadmium, the analysis revealed that older fish accumulated meaningfully higher levels in their liver tissue. At the same time, the ecological group and the helminth burden did not have a substantial impact. Several studies confirm the role of age in metal bioaccumulation (e.g., Has-Schon et al. 2015; Ansel and Benamar 2018). Under natural conditions, the age of fish primarily affects the concentration of metals in their bodies due to the duration of exposure. This is evident from current results showing that cadmium levels in the liver are related to the age of the fish.

## Electronic supplementary material

Below is the link to the electronic supplementary material.

Supplementary Material 1
