# Supplementary material for: Heavy Metal Accumulation in Freshwater Fish: The Role of Species, Age, Gender, and Parasites
Source: Bull Environ Contam Toxicol. 2025 Jun 10;114(6):92. doi: 10.1007/s00128-025-04068-z (PMC12152064; doi:10.1007/s00128-025-04068-z)
Supplement: Supplementary file 1 — Supplementary Material 1 [file 128_2025_4068_MOESM1_ESM.docx]

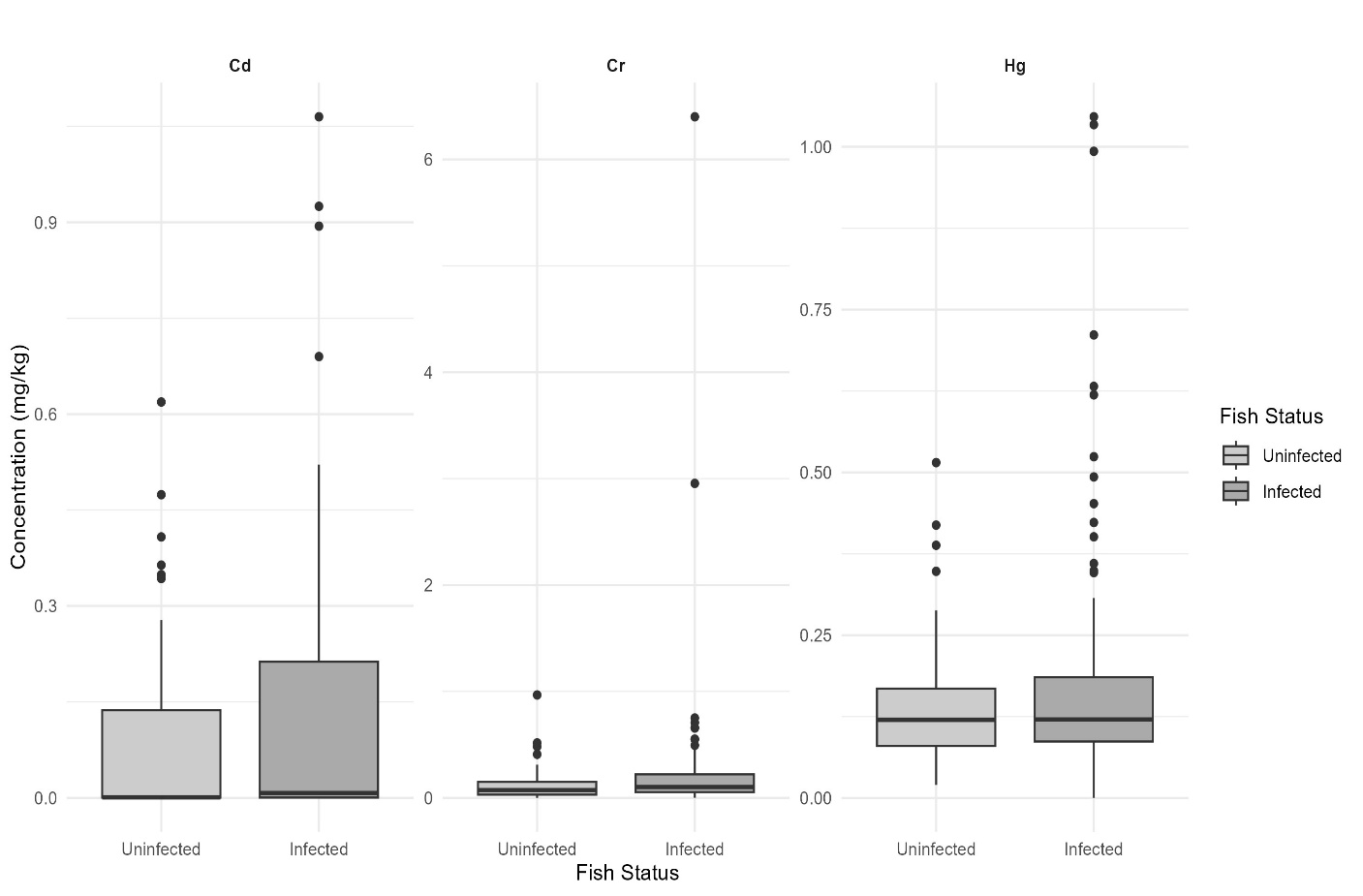


Figure S1: A descriptive comparative analysis of heavy metal concentrations (mg.kg^-1^ w. wt) across infected (dark grey) and uninfected fishes (light grey).
